# Morbidity patterns of persons in prisons compared to the general Hungarian population between 2017 and 2019

**DOI:** 10.1016/j.puhip.2025.100639

**Published:** 2025-07-02

**Authors:** P. Fadgyas-Freyler, Á. Münnich, J. Molnár, K. Kósa

**Affiliations:** a Department of Health Policy, Institute of Social and Political Sciences, Corvinus University, Budapest, Hungary; b Department of Behavioural Sciences, Faculty of Medicine, University of Debrecen, Debrecen, Hungary

**Keywords:** Prison health, Morbidity groups, Relative risk of health care utilisation, Health inequalities

## Abstract

**Objectives:**

Prison population around the globe tends be among the most marginalized population groups. Many of these persons struggle with numerous health problems, primarily with infectious diseases and mental disorders. Our goal was to analyse morbidity of prisoners compared to age- and gender-matched controls.

**Study design:**

Case-control study based on administrative data of healthcare utilisation in Hungary between 2017 and 2019.

**Methods:**

Patient numbers by ICD-10 chapters and tabulation lists of diagnosis (morbidity groups) were obtained from the government-funded single payer health insurance institute. Convicts were compared to controls matched by age and gender in a 5:1 ratio.

**Results:**

The leading causes of healthcare utilisation of prisoners compared to controls by ICD-10 chapters were mental and behavioural disorders and external causes of morbidity, but the relative risk of healthcare use due to infectious diseases was not elevated. When investigating data at a more detailed level, that is, by morbidity groups, the relative risk of healthcare utilisation due to 19 out of a total of 299 morbidity groups was significantly, more than 3 times higher among prisoners compared to controls, and the first five highest relative risks were all due to external causes of injury.

**Conclusion:**

The leading causes of morbidity among persons in prisons based on health care utilisation seems to be somewhat different from morbidity patterns identified by other methods such as clinical interviews, screening or estimations. Causes of health care utilisation both at ICD chapter and morbidity group-level is one of several relevant indicators of the health care needs of prison populations that uncovers the groups of most severe maladies among them. However, other sources of data should also be taken into account for the development of effective preventive and rehabilitative interventions. Follow-up of prisoner patients treated in health care would also be recommended to aid rehabilitation.

## 1 Introduction

The “Health for All” concept that was first issued by the World Health Organization (WHO) in 1978 and renewed in 1995 brought the health of certain marginalized groups into the focus of public health. One emerging group was that of prisoners whose specific health problems had been gradually revealed in the past decades.

### 1.1 Infectious diseases

US authors in 1993 summarized and reported the prevalence of infectious conditions such as human immunodeficiency virus (HIV), hepatitis B (HBV), and the most important sexually transmitted infections (STI such as e.g. gonorrhoea, chlamydia, syphilis). They found the prevalence of HIV-1 infection ranging between 0.2 and 26 %, tuberculosis (TB) between 12.7 and 27 %, HBV between 19 and 46.8 %, gonorrhoea between 0.4 and 18.4 %. They also emphasized the public health role that the criminal justice system can play by detecting, treating and controlling communicable diseases not only in prisons but in large urban communities as well [1].

The Centers for Disease Control and Prevention (CDC) of the USA recommended HBV vaccination for all non-vaccinated persons in long-term correctional facilities in its guidelines for sexually transmitted diseases treatment in 2002 [2]. Recognizing the magnitude of the problem, the most recent CDC guidelines for STI treatment (2021) extended its applicability to any patient care setting that serves at-risk persons, including correctional health care settings [3].

Other authors also found STIs to be highly prevalent among incarcerated persons around the world: pooled prevalence estimates for chlamydia were 5.75 % (95 % CI 5.01, 6.48) for men, 12.31 % (95 % CI 10.61, 14.01) for women; for gonorrhoea 1.4 % (95 % CI 1.09, 1.70) in men and 5.73 % (4.76, 6.69) in women; and for syphilis 2.45 % (95 % CI 2.08, 2.82) in men and 6.10 % (95 % CI 4.75, 7.46) in women, respectively [4].

The European CDC (ECDC) compiled a systematic review on active case finding of communicable diseases in prisons in 2017 which found that active case finding tends to focus on HBV and HCV, HIV, STIs and TB. The review called for more research to assess the effectiveness of interventions such as uptake of testing and active case finding in prisons [5].

### 1.2 Mental health

Regarding the disease patterns, increased psychiatric morbidity among prisoners was found by one of the earliest surveys undertaken in England and Wales in 1997 [6]. The survey relied on lay and clinical interviews with 5.2 % of the total prison population. The sample was stratified by gender and status (sentenced/remand). The prevalence of any personality disorder was the highest with 78 % for male remand, 64 % for male sentenced and 50 % for female prisoners, antisocial personality disorder being the most prevalent personality disorder. The proportion of prison population in various groups scoring at or above the threshold of neurotic symptoms ranged from 39 % to 75 % as opposed to 12 % of men and 18 % of women in a nationwide household survey. The lifetime prevalence of suicide attempt was 46 % among remand males, whereas self-harm without the intention of suicide ranged from 5 % for remand males to 10 % for sentenced females. The prevalence of hazardous drinking assessed by the Alcohol Use Disorders Identification Test (AUDIT) was at least 58 % among male and at least 36 % among female prisoners, while the lifetime prevalence of drug use was at least 81 % among male and 77 % among female prisoners.

Steadman et al. found that 14.5 % of male and 31.0 % of females in some jails of two US states qualified for a diagnosis of serious mental illness by using the Structured Clinical Interview for DSM-IV (SCID) [7].

Age- and sex-adjusted prevalence of any mental disorders among offenders was almost 1.5 times higher compared to nonoffenders between 2007 and 2012 with mood and anxiety disorders having the highest, substance use disorders the second highest prevalence in Manitoba, Canada [8].

Swedish authors assessing imprisoned male offenders between 2010 and 2012 and following them until 2017 found that they more often received psychiatric diagnoses and psychotropic drugs compared to the general population. Prior psychiatric contact, foster home placement, low intellectual functioning and persistent offending were found to be important risk factors for psychiatric healthcare utilisation [9].

Suicide as a major risk outcome of mental disorders was also found to be elevated among persons in prisons. Fazel et al. found at least three times higher relative rate of suicide in a large database of prison suicides gathered in 12 countries [10]. Based on 10-year data from the Annual Penal Statistics of the Council of Europe, Rabe also confirmed that prison suicide rate was on average seven times higher than in the general population [11].

A seven-year observational prison study in an Italian region [12] detected a suicide rate of 1.12 per 1000 persons in prisons – as opposed to a mean crude rate of 7.2 per 100 000 persons in the general population [13].

Gottfried et al. concluded via a literature review that mental disorders are more common among criminals than in the general population [14].

Favril et al. conducted an umbrella review based on 17 meta-analyses published between 2002 and 2023 and found that mental disorders (primarily major depression, alcohol use disorder and drug use disorder) as well as infectious diseases (primarily HCV and HBC as well as HIV and TB) comprised the leading causes of morbidity among prisoners albeit with significant differences by sex and country income level [15].

Regarding the external causes of morbidity, self-harm is a leading cause in prison populations. Based on 35 studies from 20 countries including more than 660 thousand prisoners, Favril et al. described that 3.8 % of them self-harmed while incarcerated. Regarding the risk factors of self-harm the strongest associations were found with recent suicidal ideation, lifetime history of suicidal ideation, previous self-harm, any current psychiatric diagnosis, solitary confinement, disciplinary infractions and experiencing victimization in prison [16].

Most of the described research was based on clinical interviews, estimations and on-site testing. Our goal was to analyse the most important causes of morbidity by ICD-10 morbidity groups with the help of administrative data of health care utilisation among those in prisons compared to age- and gender-matched controls not in prisons in Hungary between 2017 and 2019.

## 2 Methods

### 2.1 Source of data

Administrative (claims) data were provided by the 10.13039/100014477National Health Insurance Fund, the government-funded single payer of health care in Hungary (responsible for roughly 10 million persons). Data such as diagnosis, date of interventions, etc. are usually set by medical professionals (doctors, nurses) during the treatment process and are later (monthly) transferred to the Insurance Fund within the framework of the reimbursement process based on a fee-for-service methodology. Prescribing data are transferred to the Fund from the pharmacies with diagnosis codes set by the doctors as well. From these different claim databases (separate for primary and secondary outpatient care as well as inpatient care and pharmacies, etc.) a single database was created within the Fund with aggregated patient numbers for case and control groups for all morbidity groups. In order to align with data privacy laws according to which no personal identification of patients is allowed any morbidity categories with less than 10 patients were provided without the exact number of patients.

### 2.2 Study population

All incarcerated criminal offenders in Hungary are entitled to use publicly funded healthcare, and incarcerated status is a source of entitlement to health services. For the period between 2017 and 2019 (that is, before the Covid-19 pandemic) 31 137 adults (over 21 years of age) incarcerated for any reason were selected and compared to a control group (N = 155 685 persons) comprised of nonoffenders selected from the whole population. Cases (those in prisons) and controls (those not in prisons) were matched by age and gender. Controls were fitted to cases in a 5 to1 ratio (5 nonoffenders were selected for every one offender). This way, the entire study population included 186 822 persons. Average age was 37 years, 4,5 %–5 % were females, thus demographic characteristics of the study population align with typical European numbers [17].

ICD-10 diagnosis and tabulation list for morbidity (morbidity groups) as specified by the WHO [18], as well as data on age, gender, incarcerated status (yes/no), year of healthcare utilisation, patient numbers and direct health care costs separately for a) diagnostic procedures (laboratory and medical imaging), b) outpatient specialist care, c) inpatient care, d) prescribed medication, e) dental treatment, f) dialysis, and g) any other medical care were provided in Excel tables.

### 2.3 Statistical analysis

Descriptive and analytical statistics were calculated using Excel 2016 and Wolfram Mathematica 14.0. Relative risk of health care utilisation by ICD-10 codes and morbidity groups were calculated using patient numbers of the incarcerated and non-incarcerated (control) groups.

### 2.4 Limitations

The advantage of our methodology is the size of the population level dataset, but this also has some handicaps that we do not wish to conceal. First of all: all data are coming from the publicly funded healthcare system so that private healthcare is not included. However, in the study period before the COVID pandemic private providers played a smaller supplementary role in very specific parts of the Hungarian healthcare system such as high-cost diagnostics (e.g. PET/CT) or specific surgeries like hip replacements, etc. This might by a bias, but our scope lies elsewhere. Though prison hospitals exist, normal public clinics will provide care for any other problems not treatable there. A second possible shortfall of our methodology is the lack of socio-economic data of the individuals. As persons in prison have lower SES our results would be much more striking if the two groups could have been also matched regarding this matter. Unfortunately, the Fund database contains no indicators of individual income, educational level or so, so that this was not possible.

## 3 Results

First, we examined the relative risk of healthcare utilisation due to diagnoses assigned by ICD chapters. The number of prisoners treated in a given year was compared to fitted controls by diagnostic chapter. As it is shown in Table 1, the relative risk of healthcare utilisation due to ‘Mental and behavioural disorders’ (Chapter V) was the highest, at least 4 times higher compared to the controls in all 3 years.

**Table 1:** Relative risk of healthcare utilisation due to diseases by ICD chapter among prisoners compared to an age- and gender-matched control group of the general population.

ICD-10		2017			2018			2019		
Chapter	Title	RR	CI-lower	CI-upper	RR	CI-lower	CI-upper	RR	CI-lower	CI-upper
V	Mental and behavioural disorders	**4.29**	4.19	4.39	**4.17**	4.08	4.27	**4.07**	3.98	4.17
XX	External causes of morbidity and mortality	**2.38**	2.16	2.62	**2.54**	2.32	2.79	**2.84**	2.58	3.12
XXII	Codes for special purposes	**2.61**	2.58	2.65	**2.41**	2.38	2.45	**2.30**	2.27	2.34
XIX	Injury, poisoning and certain other consequences of external causes	**1.78**	1.75	1.81	**1.79**	1.75	1.82	**1.71**	1.68	1.75
XVIII	Symptoms, signs and abnormal clinical and laboratory findings, not elsewhere classified	**1.54**	1.51	1.58	**1.55**	1.52	1.58	**1.48**	1.45	1.51
XVI	Certain conditions originating in the perinatal period	1.46	0.72	2.96	1.25	0.59	2.65	1.29	0.67	2.48
VI	Diseases of the nervous system	**1.21**	1.15	1.27	**1.27**	1.21	1.33	**1.30**	1.25	1.37
XI	Diseases of the digestive system	0.99	0.99	1.03	1.03	1.01	1.05	1.06	1.04	1.07
XV	Pregnancy, childbirth and the puerperium	0.97	0.90	1.18	0.95	0.92	1.21	1.09	0.95	1.24
XII	Diseases of the skin and subcutaneous tissue	0.95	1.02	1.08	0.95	1.02	1.09	0.98	0.99	1.06
IX	Diseases of the circulatory system	0.93	1.05	1.10	0.93	1.05	1.10	0.93	1.06	1.10
XIII	Diseases of the musculoskeletal system and connective tissue	0.89	1.09	1.14	0.92	1.06	1.11	0.88	1.11	1.16
XXI	Factors influencing health status and contact with health services	0.86	1.13	1.20	0.88	1.11	1.18	0.93	1.04	1.10
I	Certain infectious and parasitic diseases	0.89	1.08	1.17	0.83	1.15	1.25	0.93	1.03	1.11
X	Diseases of the respiratory system	0.74	1.32	1.37	0.73	1.34	1.39	0.73	1.34	1.39
XIV	Diseases of the genitourinary system	0.65	1.48	1.60	0.66	1.45	1.57	0.73	1.32	1.43
VIII	Diseases of the ear and mastoid process	0.67	1.42	1.59	0.66	1.43	1.61	0.66	1.42	1.60
IV	Endocrine, nutritional and metabolic diseases	0.62	1.55	1.67	0.65	1.47	1.58	0.67	1.45	1.55
VII	Diseases of the eye and adnexa	0.60	1.58	1.74	0.56	1.69	1.86	0.64	1.49	1.63
III	Diseases of the blood and blood-forming organs	0.54	1.68	2.05	0.53	1.70	2.06	0.61	1.50	1.80
XVII	Congenital malformations, deformations and chromosomal abnormalities	0.47	1.71	2.61	0.40	2.00	3.20	0.39	2.05	3.24
II	Neoplasms	0.37	2.51	2.93	0.39	2.39	2.78	0.44	2.12	2.44

Note: Values in **bold** show significantly increased risk among prisoners compared to the control.

The next highest relative risk of healthcare utilisation inconsistently varied during 2017–2019 between ‘External causes of morbidity and mortality’ (Chapter XX) and ‘Codes used for special purposes’ listed in Chapter XXII. Additionally, significantly increased relative risks of healthcare utilisation for prisoners were also found in all 3 years in relation to ‘Injury, poisoning and other consequences of external causes’ (Chapter XIX); ‘Symptoms, signs and abnormal findings not elsewhere classified’ (XVIII); and ‘Diseases of the nervous system’ (Chapter VI). The relative risks of healthcare utilisation due to diseases coded in other chapters of ICD 10 are no different from those of the control population as shown by the nonsignificant relative risks due to the remaining Chapters in Table 1.

The yearly relative risks of healthcare utilisation by ICD-10 Chapters were found to be strongly correlated by Pearson correlation with correlation coefficients of 0.987 or higher (not shown) among the three investigated years. This proves that relative risks were consistent between 2017 and 2019.

**Fig. 1:**
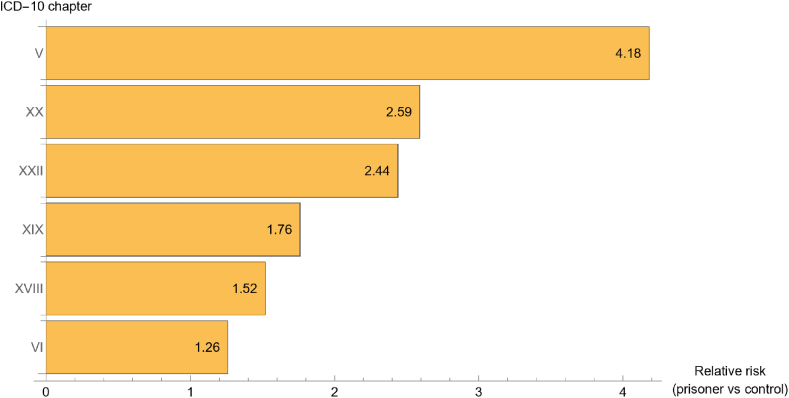
Significantly increased mean relative risk of healthcare utilisation between 2017 and 2019 due to diseases by ICD chapter among prisoners vs an age- and gender-matched control group from the general population.

We have also calculated the mean relative risks for the first six ICD chapters with highest relative risk. By averaging the relative risks of each investigated year (2017, 2018, 2019), the rank order of the mean relative risks for the whole study period was the same as the yearly rank orders: the highest mean relative risk (more than 4 times higher among prisoners compared to the age- and gender-matched control population) of healthcare utilisation was due to ‘Mental disorders’ (Chapter V); the second highest mean relative risk was due to ‘External causes of morbidity and mortality’(Chapter XX); the third highest due to ‘Codes listed for special purposes’ (Chapter XXII, listing diseases of uncertain causes and drug resistance). The rank order of the other mean relative risks was the same as found when each year was analysed separately: 4th: ‘Injury, poisoning and other consequences of external causes’ (Chapter XIX); 5th: ‘Symptoms, signs and abnormal findings not elsewhere classified’ (XVIII); 6th: ‘Diseases of the nervous system’ (Chapter VI) (Fig. 1).

Next, we examined the relative risk of healthcare utilisation by special tabulation lists or morbidity groups (N = 299) specified by the WHO.

The number of prisoners treated because of diagnoses belonging to specific morbidity groups in each year were compared to patient number of the control group with the same morbidity. Table 2 shows those 19 morbidity groups (out of a total of 299 morbidity groups) for which the relative risk of healthcare utilisation was considerably (2.5–40 times) higher among prisoners compared to controls in all three years. Interestingly, the first five morbidity groups with highly increased (over five times higher) relative risks of healthcare utilisation were all related to ‘External causes of injury’ and not to any ‘Mental and behavioural disorders’ as would have been expected based on the relative risks of the higher aggregation level by ICD chapters shown in Table 1. Of the 19 morbidity groups, 10 belong to ‘External causes of morbidity’ (with green background), 4 are ‘Mental and behavioural disorders’ (designated with orange background), and only 3 refer to ‘Infectious diseases’ (with yellow background).

**Table 2:**
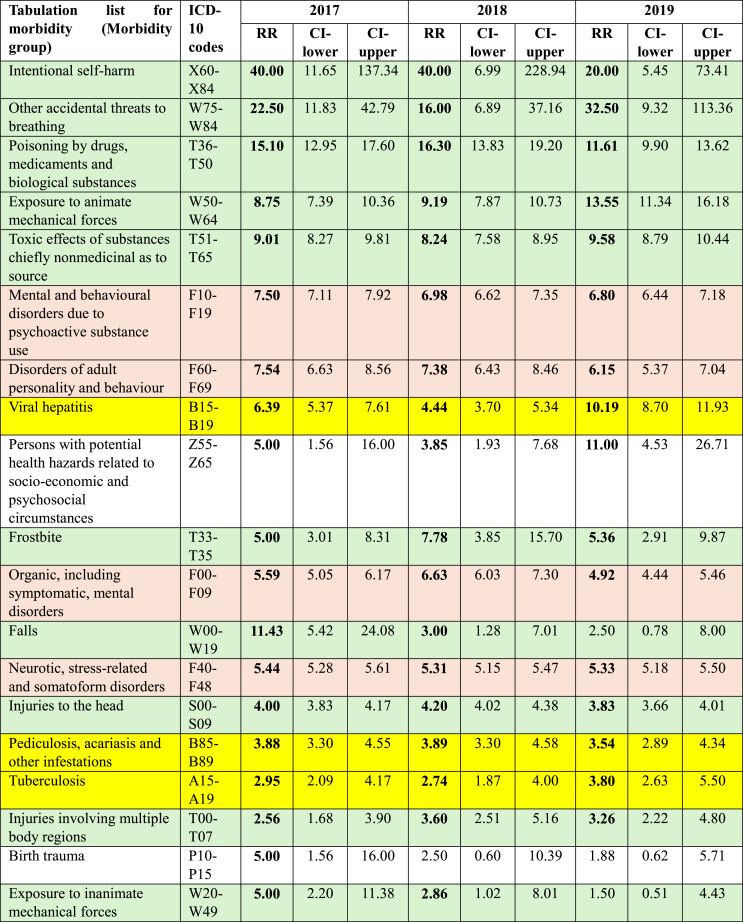
Relative risk of healthcare utilisation by morbidity group among prisoners compared to an age- and gender-matched control group of the general population.

Note: Values in **bold** show significantly increased risk among prisoners compared to controls.

‘Health hazards related to socioeconomic and psychosocial circumstances’ caused significantly increased risk of healthcare utilisation among prisoners in each investigated year. ‘Birth trauma’ as a cause of increased relative risk of healthcare use for prisoners was significant only in 2017 (Table 2).

The yearly relative risks of healthcare utilisation by morbidity groups were found to be strongly correlated by Pearson correlation with correlation coefficients of 0.810 or higher (not shown) among the three investigated years. This proves that relative risks by morbidity groups were also consistent between 2017 and 2019.

**Fig. 2:**
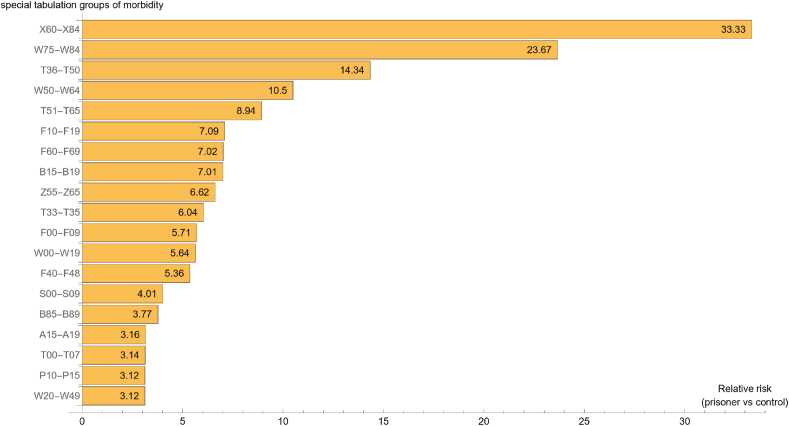
Significantly increased mean relative risk of healthcare utilisation between 2017 and 2019 by morbidity groups among prisoners vs age- and gender-matched control group of the general population.

We have also calculated the mean relative risk of healthcare utilisation for prisoners compared to age- and gender-matched controls for each of the 19 morbidity groups which were scoring high between 2017 and 2019. These mean values of relative risks can be seen in Fig. 2. The rank order of the mean relative risk by morbidity groups was the same as the yearly rank order. That is, the highest mean risks (RR ≥ 8) of healthcare utilisation among prisoners compared to controls were due to ‘External causes of morbidity’, with ‘Intentional self harm’ standing out with a mean relative risk over 30.

## 4 Discussion

We investigated the patterns of morbidity by ICD-10 morbidity groups based on health care utilisation by adults in prisons compared to age- and gender-matched controls using administrative data of health care financing in Hungary between 2017 and 2019. When analysing causes of utilisation by ICD chapters, we found – similarly to other studies – that leading causes were mental and behavioural disorders and external causes of morbidity, with mental diseases absolutely dominating the landscape of health problems. However, on that aggregation level infectious diseases, another prevalent group of diseases among prisoners was unexpectedly not higher than in the control group.

When investigating the causes of health care utilisation in a lower level of aggregation (morbidity groups), a different pattern was found. The greatest differences, at least 8 times higher risk of utilisation were all due to ‘External causes of injury’, be it mechanical or chemical, intentional or accidental, with intentional self-harm being the highest risk. Regarding the latter, it is worth noting that according to the Annual Penal Statistics of 2022 of the Council of Europe, the suicide rate of persons in prisons in 2021 was very low in Hungary, meaning that the score was more than 25 % lower than the European median value [17]. Self-harm (or non-suicidal self-injury according to the DSM-5) is a deliberate act of causing harm to oneself that may present as cutting, biting, burning, consuming drugs with a self-aggressive intent, etc. [19] Though intentional or deliberate self-harm (DSH) does not equal suicidal ideation or intent, a recent study found that more than half of those with DSH had significant suicide risk, and 99.1 % of adolescents with suicide attempts also reported DHS [20] Since many of the known risk factors of intentional self-harm such as childhood abuse, depression, anxiety, bullying, substance use etc [21]. have been known to affect persons in prisons, and being in prison generates additional risk factors for suicide attempts [22], it would seem reasonable to carry out a mental health evaluation of all those in prisons who need health care treatment due to self-harm.

At this more detailed level, specific infections have also emerged as worthy of attention, such as TB, hepatitis and infections with relation to parasites. Unfortunately, we could also see that the prison population seem to be more prone to acquire infections that are more resistant to treatments. This may be due to the way of living before coming to prison or proper hygiene once in prison or a combination of both. Nevertheless, efforts should be made to improve proper hygiene, prevention of infections (eventually with systematic screening and automatic vaccination by entering the correctional facilities), and health education. With regard to infections we emphasise that HIV infection did not have an elevated risk for the prison population, and this is in alignment with the national trend of low incidence of HIV infection and low prevalence of AIDS [23].

Our data draw attention to the fact that health care utilisation of prisoners - specified by major diagnostic categories - are dominated by categories of mental disorders.

A unique strength of our study derives from the fact that health care institutes for prisoners are part of the nationwide system of health care in Hungary with a single payer. This fact enabled us to select an age- and gender-matched control group from the general Hungarian population of adults.

Health problems, especially mental health problems of prisoners have been recognized not only as a topic for public health but also as a topic for wider social debate. The optimal care of psychiatric patients has been contentious since the mid-20th century when de-institutionalization of psychiatric patients gained traction in developed countries, followed by reports that these measures had resulted in many deinstitutionalized patients entering and increasing the prison and/or the homeless population [24,25]. Though a more recent systematic review did not support these findings [26], the increased prevalence of mental disorders among and the marginalized status of prisoners has been widely acknowledged [[27], [28], [29]].

Public health has come a long way in terms of improving the plight of prisoners. WHO compiled a book with suggestions to improve the health of prisoners, focusing on bloodborne viruses, tuberculosis prevention, mental and oral health [30]. A more recent publication operationalized the WHO prison health framework and called – among others – for measuring the performance of prison health systems [31] – an aspect of health service delivery to which the monitoring of the use of specialized medical care by persons in prison could contribute by revealing gaps in the provision of preventive and/or primary care.

Another guideline of the CDC in the USA on STI treatment is applicable to any health care setting including correctional health care facilities [3]. However, a recent survey of more than 1000 jails in the USA found that, contrary to recommendations only 7 % of jails test individuals for HIV infection at admission. Recommendations for pre-exposure prophylaxis of HIV, testing for HCV and TB during jail admission were also not followed [32].

The competent authority in Europe (ECDC) has issued guidelines for prisons regarding active case finding of communicable diseases [33] and preventing blood-borne viruses [34].

A recent publication from Latin-American authors reiterated the need to strengthen the surveillance and treatment of TB not only among those who are incarcerated but also in their wider communities after release [35].

The Council of Europe issued a White Paper on the management of persons with mental health disorders in prisons [36]. The Paper makes a number of recommendations that align with our own, especially the one for steps to take for the detection, prevention and proper treatment of mental disorders in prisons.

Our findings allude to the possibility that various sources of data on prison health highlight overlapping but not identical sets of health problems: interviews and surveys conducted among prisoners can identify mental problems, risks of infectious diseases and socio-economic risks, whereas data on health care utilisation reveal a slightly different set of health problems by which external causes of morbidity requiring immediate specialized medical care seem to dominate. Interestingly, a nested case-control study conducted for 14 years in Australia found an increased risk of external causes of mortality – dominantly from drug overdose, suicide, transport accidents and violence – among those released from prison [37]. This raises the possibility that offenders in prisons have a better chance to survive external causes of injury especially if these require immediate medical care than offenders out of prisons. Some external causes of mortality, especially drug overdose, suicide and violence are related to underlying mental health problems such as mood and/or conduct disorders and personality disorders. Thus, the question arises whether mental health of the prison population is properly investigated, whether appropriate preventive and curative services are provided for them. Such services would serve not only prisons but also society as a whole.

Despite of the efforts from the research community and international health agencies, health status of prisoners remains a public health concern. Effective interventions should target major risk factors utilising the advantage of prison settings, meaning that strict regulation and schedules might be helpful to provide for those who need these health interventions the most.

## Ethical approval

We declare that no authors received any financial funding for this work.

## Declaration of competing interest

The authors declare that they have no known competing financial interests or personal relationships that could have appeared to influence the work reported in this paper.
